# Favoring Expression of Yak Alleles in Interspecies F1 Hybrids of Cattle and Yak Under High-Altitude Environments

**DOI:** 10.3389/fvets.2022.892663

**Published:** 2022-06-30

**Authors:** Shi-Yi Chen, Cao Li, Zhihao Luo, Xiaowei Li, Xianbo Jia, Song-Jia Lai

**Affiliations:** ^1^Farm Animal Genetic Resources Exploration and Innovation Key Laboratory of Sichuan Province, Sichuan Agricultural University, Chengdu, China; ^2^Longri Breeding Farm of Sichuan Province, Hongyuan, China

**Keywords:** allele expression, Nanopore long-read RNA-seq, transcriptome, cattle, yak

## Abstract

Both *cis*- and *trans*-regulation could cause differential expression between the parental alleles in diploid species that might have broad biological implications. Due to the relatively distant genetic divergence between cattle and yak, as well as their differential adaptation to high-altitude environments, we investigated genome-wide allelic differential expression (ADE) in their F1 hybrids using Nanopore long-read RNA-seq technology. From adult F1 hybrids raised in high-altitude, ten lung and liver tissues were individually sequenced for producing 31.6 M full-length transcript sequences. Mapping against autosomal homologous regions between cattle and yak, we detected 17,744 and 14,542 protein-encoding genes expressed in lung and liver tissues, respectively. According to the parental assignments of transcript sequences, a total of 3,381 genes were detected to show ADE in at least one sample. There were 186 genes showing ubiquitous ADE in all the studied animals, and among them 135 and 37 genes had consistent higher expression of yak and cattle alleles, respectively. Functional analyses revealed that the genes with favoring expression of yak alleles have been involved in the biological progresses related with hypoxia adaptation and immune response. In contrast, the genes with favoring expression of cattle alleles have been enriched into different biological progresses, such as secretion of endocrine hormones and lipid metabolism. Our results would support unequal contribution of parental genes to environmental adaptation in the F1 hybrids of cattle and yak.

## Introduction

Within the *Bos* genus, the two species of cattle (*Bos taurus*) and yak (*B. grunniens*) evolutionally diverged ~4.9 million years ago (1). However, there is no reproductive barrier to hybridization between cattle and yak regarding both reciprocal crosses, although the male F1 hybrids are sterile that may be possibly resulting from spermatogenesis failure (2). Most modern cattle breeds have evolved into high production performance in terms of economically important traits, whereas yak is well adapted to high-altitude environments due to long-term adaptive evolution (3). Meanwhile, both physiological fitness and production performance of cattle will considerably decrease when residing above 2,500 m altitude (4). In phenotype, therefore, the interspecies F1 hybrids of cattle and yak have the significantly improved milk yield and growth, as well as great physiological adaptation to high-altitude environment. Yet, the underlying genomic basis remains poorly characterized.

For autosomal non-imprinted genes in diploid species, the two allelic copies that are inherited from parents could be differentially expressed, which is termed allelic differential expression (ADE) and has broad biological implications as an important regulatory variation (5, 6). Obviously, the most extreme case of ADE is monoallelic expression that was observed to be widespread (7, 8). The genome-wide landscape of ADE in humans and diverse species (such as cattle, chicken, and rice) have been investigated comprehensively using the large numbers of single nucleotide polymorphisms (SNPs) obtained by oligonucleotide array and high-throughput sequencing technologies (8–12). The degree of genetic differentiation between two parents would influence detection ability of allelic expression levels in offspring. The genomic heterozygosity of F1 hybrids of cattle and yak was recently estimated to be ~1.2%, which is higher than cross-breed hybrids of taurine and indicine cattle (13). Therefore, the highly heterogeneous diploid genome of F1 hybrids of cattle and yak provides us a great opportunity to investigate ADE, especially in such case that two parents have differential production performance and adaptation to the local environments. Using the short reads of transcriptome sequencing, Wang et al. (14) first reported hundreds of genes (~5% of all analyzed genes) showing ADE in the liver and ear tissues of F1 hybrids of cattle and yak.

Second-generation RNA sequencing technologies (i.e., short-read RNA-seq) have been becoming the most widely used approach for studying gene expression (15). However, two analytical methodological issues should be taken into consideration when studying ADE based on short sequencing reads. The first issue is mapping bias of short reads against reference genome, which would lead to an over-estimation of reference allele in relative to alternate allele (16, 17). Second, allelic expression is indirectly quantified by counting the distinguishable short reads instead of full transcript sequences, mainly as it is difficult to accurately assemble allele-specific transcripts (18). Recently, the long-read RNA sequencing technologies (i.e., long-read RNA-seq) have been increasingly used for quantifying allelic expression (19, 20). In this study, we employed single-molecule long-read sequencing technology from Oxford Nanopore Technologies (ONT) to investigate global gene expression in F1 hybrids of cattle and yak. After quantifying allelic expression based on the full transcript sequences, the genes with ADE and the possibly involved biological implications were analyzed. These results are expected to improve our understanding of parental contribution to F1 hybrids of cattle and yak regarding the environmental adaptation and production performance.

## Materials and Methods

### Animals and Sample Preparation

**Figure 1:**
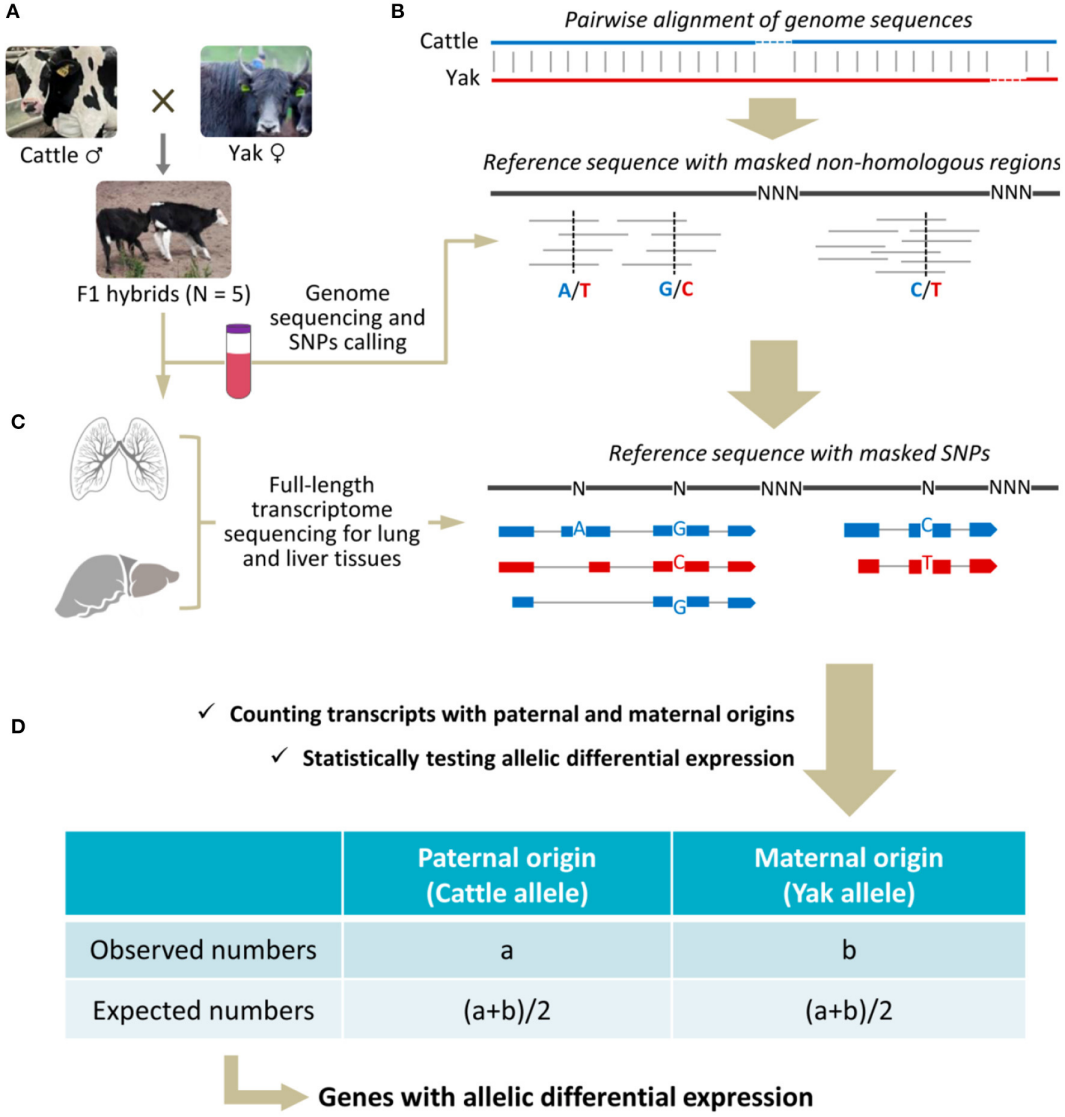
Schematic illustration of sample collection **(A)**, preparation of reference genome sequences **(B)**, sequencing and mapping of the long reads **(C)**, and quantification and statistical testing of parental allelic expression **(D)**.

Five adult (~5 years of age, two males and three females) and healthy F1 hybrids of cattle and yak were randomly included in this study (Figure 1A), which had been raised in the Longri Breeding Farm of Sichuan Province at an altitude of ~3,000 m. The five animals were individually sequenced as biological replicates. After being slaughtered with head-only electrical stunning, the tissue samples of lung and liver were immediately collected and frozen in liquid nitrogen. In parallel, the peripheral blood was collected and used for genome sequencing. Total RNA was extracted using RNASimple Total RNA Kit (Tiangen Biotech, Beijing, China) following manufacturer's instructions. RNA concentration and RNA integrity number (RIN) were analyzed using Nanodrop 2000C (Thermo Fisher Scientific, Waltham, USA) and Agilent 2100 Bioanalyzer (Agilent Technologies, Santa Clara, USA), respectively (Supplementary Table S1). Genomic DNA was isolated using TIANamp Genomic DNA Kit (Tiangen Biotech, Beijing, China) according to manufacturer's instructions.

### RNA and DNA Sequencing

For transcriptome sequencing, 1 μg of total RNA was used for preparing libraries with cDNA-PCR Sequencing Kit (SQK-PCS109, Oxford Nanopore Technologies). In brief, the full-length cDNAs were enriched using template switching activity of reverse transcriptase. The PCR adapters were directly added to both ends of the first-strand cDNAs. PCR amplification was performed with 14 circles using LongAmp Tag (NEB), and then PCR products were subjected to ONT adaptor ligation using T4 DNA ligase (NEB). The Agencourt XP beads was used for DNA purification according to official protocol. The final cDNA libraries were added to FLO-MIN109 flowcells and run on PromethION platform at Biomarker Technology Company (Beijing, China).

For genome sequencing, the paired-end libraries with 350 bp of insert sizes were constructed according to Illumina's protocol (Illumina, San Diego, CA, USA). In brief, 0.5 μg of genomic DNA was fragmented, end-paired and ligated to adaptors, respectively. After the P2 adapter was added, DNA fragments were fractionated and purified by PCR amplification. Finally, the sequencing libraries were sequenced using Illumina HiSeq2000 at Biomarker Technology Company (Beijing, China).

### Quality Controls of Sequencing Reads

Among raw long reads of transcriptome sequencing, full-length and non-chimeric (FLNC) transcript sequences were determined and extracted using Pychopper software with default parameters (https://github.com/nanoporetech/pychopper). The raw short reads of genome sequencing were subjected to quality filtering using fastp software (21); and low-quality reads were removed according to the three criteria: (i) containing adaptor sequences, (ii) more than five N base, or (iii) unqualified bases (Q-value <15) higher than 40% of total read length. After these quality controls, the clean reads were generated.

### Construction of Transcriptome

To avoid potential mapping bias when using either cattle or yak genome sequence as reference, one pseudo genome sequence was constructed for mapping the FLNC transcript sequences (Figures 1B,C). First, interspecies autosomal homologous regions were detected according to pairwise alignment of reference genome sequences between cattle (ARS-UCD1.2) and yak (BosGru3.0), which was performed using -asm5 module in minimap2 software (22). Herein, non-homologous genomic regions (i.e., species-specific sequences) were excluded from reference sequence to avoid falsely positive findings of ADE. Second, short sequencing reads of genome were aligned to reference sequence using BWA-MEM algorithm and default parameters in BWA software (23). The interspecies SNPs were called using bcftools (24) and masked to alleviate potential mapping bias of long reads. Third, FLNC transcript sequences were aligned to this custom reference genome sequence using minimap2 software with parameters of “-ax splice -p 0.9 -N 1” (22). To detect novel genes, the obtained mapping results of FLNC transcript sequences were compared with the known annotation information of cattle genome.

### Quantification and Allelic Differential Expression

The expression levels of paternal and maternal alleles were quantified on each genic locus and statistically tested regarding their differential expressions (Figure 1D). First, the transcript sequence was assigned to paternal or maternal origins according to distinguishable interspecies SNPs determined in previous steps. When multiple SNPs with inconsistent assignments were found in one transcript sequence, the mode rule was applied. Furthermore, the indistinguishable transcript sequences that did not contain any interspecies SNP were proportionally assigned according to other known assignments. Second, the expression levels of paternal and maternal alleles were quantified by directly counting their respective transcript sequences, and expression level was expressed as counts per million mapped reads (CPM). Finally, the differential expressions of alleles were statistically deduced using Fisher's exact test implemented in R software (25), and the directions of favoring expression were determined regarding paternal and maternal origins. Here, a gene with ADE was statistically determined as both the fold change > 2 and *P* < 0.05.

### Functional Analyses of Candidate Genes

For candidate genes of interest, functional enrichment analyses were conducted using the g:GOSt function on g:Profiler web server (26), including the target cattle datasets of Gene Ontology (GO) terms (27) and Kyoto Encyclopedia of Genes and Genomes (KEGG) pathway (28). The default parameters and methods for adjusting for multiple hypotheses testing were used with an adjusted 5% level of significance.

## Results

### Reference Genome Sequence and Interspecies SNPs

Genomic comparisons revealed that 93.92% of the autosomal region of cattle had homologous genomic sequences in yak (Supplementary Figure S1). According to the annotation information of reference genomes, there were 19,163 common protein-encoding genes located within these homologous regions, whereas cattle and yak had 1,558 and 1,669 species-specific genes, respectively. Genome sequencing of the five hybrid animals generated the clean paired-end short reads with an average depth of 15-fold coverage, from which average 19,426,851 interspecies heterozygous SNPs (± 268,073 of standard deviation) were found (Supplementary Table S2). These SNPs were evenly distributed among the 29 autosomes (Supplementary Figure S2), and about 6.5% of them were located within the annotated exons.

### Sequencing and Construction of Transcriptome

For the ten sequenced samples of both lung and liver tissues, a total of 36,017,234 Nanopore long reads were initially generated, ranging from 3,237,972 to 4,091,924 per sample (Supplementary Table S3). More than 42% of raw reads were higher than 1 Kb in length, and their average N50 length was 1,372 bp. After quality controls, average 3,157,628 FLNC transcript sequences were obtained per sample (ranging from 2,803,438 to 3,680,467). Finally, average 98% of FLNC transcript sequences were successfully aligned to our custom reference genome.

**Figure 2:**
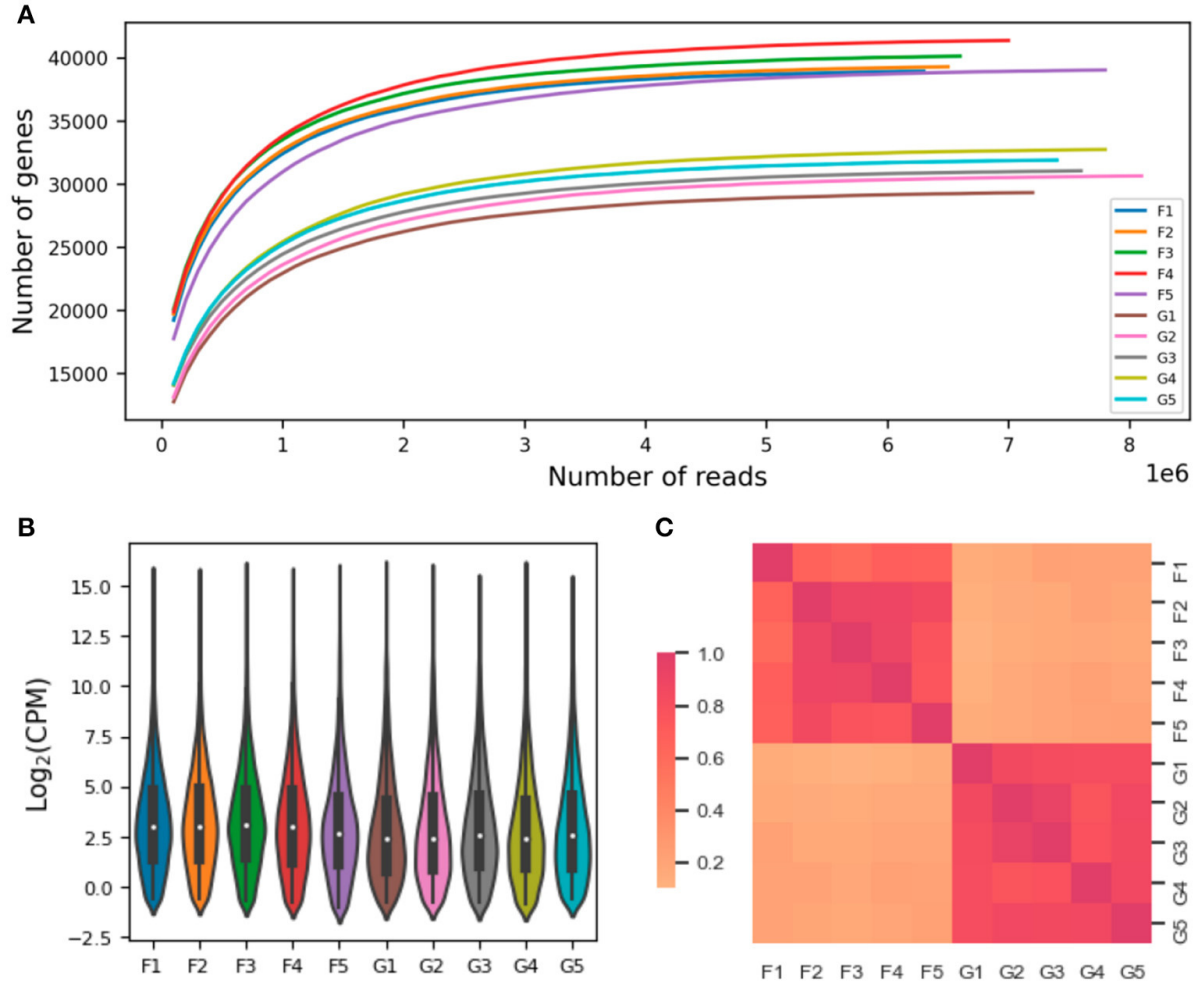
Quantification of gene expression. The individual rarefaction curves indicate relationship between the sequencing depth and number of genes observed **(A)**. The overall distribution of gene expression levels and pairwise Pearson correlations of sequenced samples are shown in **(B, C)**, respectively. The sample names were denoted by prefix of “F” and “G” for lung and liver issues, respectively; such as, the “F1” denotes the sequenced lung issue for the first animal. CPM, counts per million mapped reads.

There were 17,744 and 14,542 protein-encoding genes expressed in the lung and liver tissues, respectively. The rarefaction curve analysis revealed that the sequencing depth was enough for discovering the expressed genes (including the noncoding and pseudogenes, Figure 2A). After discarding the lowly expressed genes (i.e., with >2 mapped transcript sequences), the numbers of genes and quantified expression levels were shown in Supplementary Table S4; and the overall distribution of gene expression levels were comparable among the 10 sequenced samples (Figure 2B). The five biological replications within each tissue were demonstrated according to the pairwise Pearson correlation coefficients (Figure 2C).

### Allelic Differential Expression and Functional Analyses

**Figure 3:**
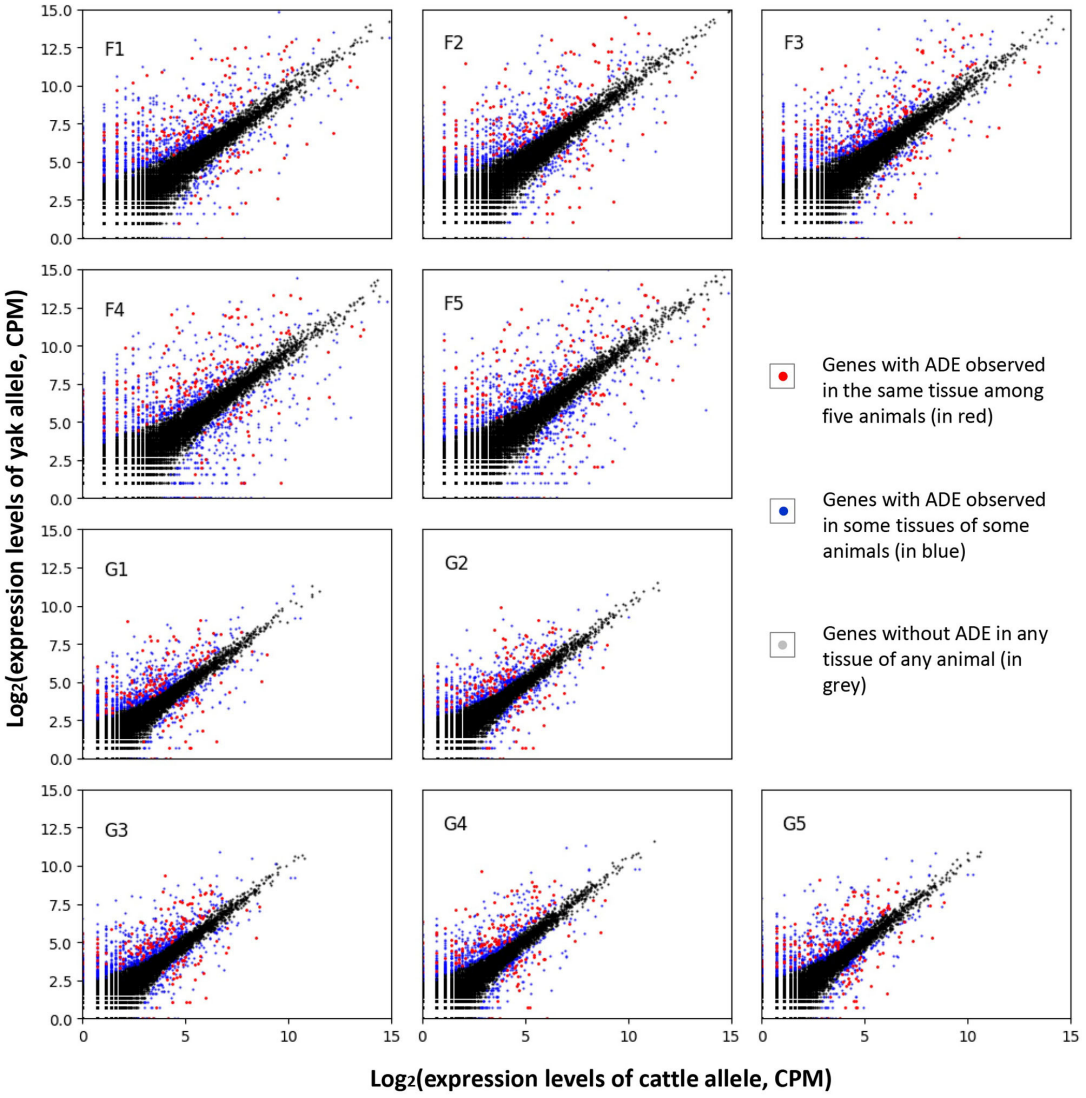
Allelic differential expression among the sequenced samples. The sample names were denoted by prefix of “F” and “G” for lung and liver issues, respectively; such as, the “F1” denotes the sequenced lung issue for the first animal. CPM, counts per million mapped reads.

**Figure 4:**
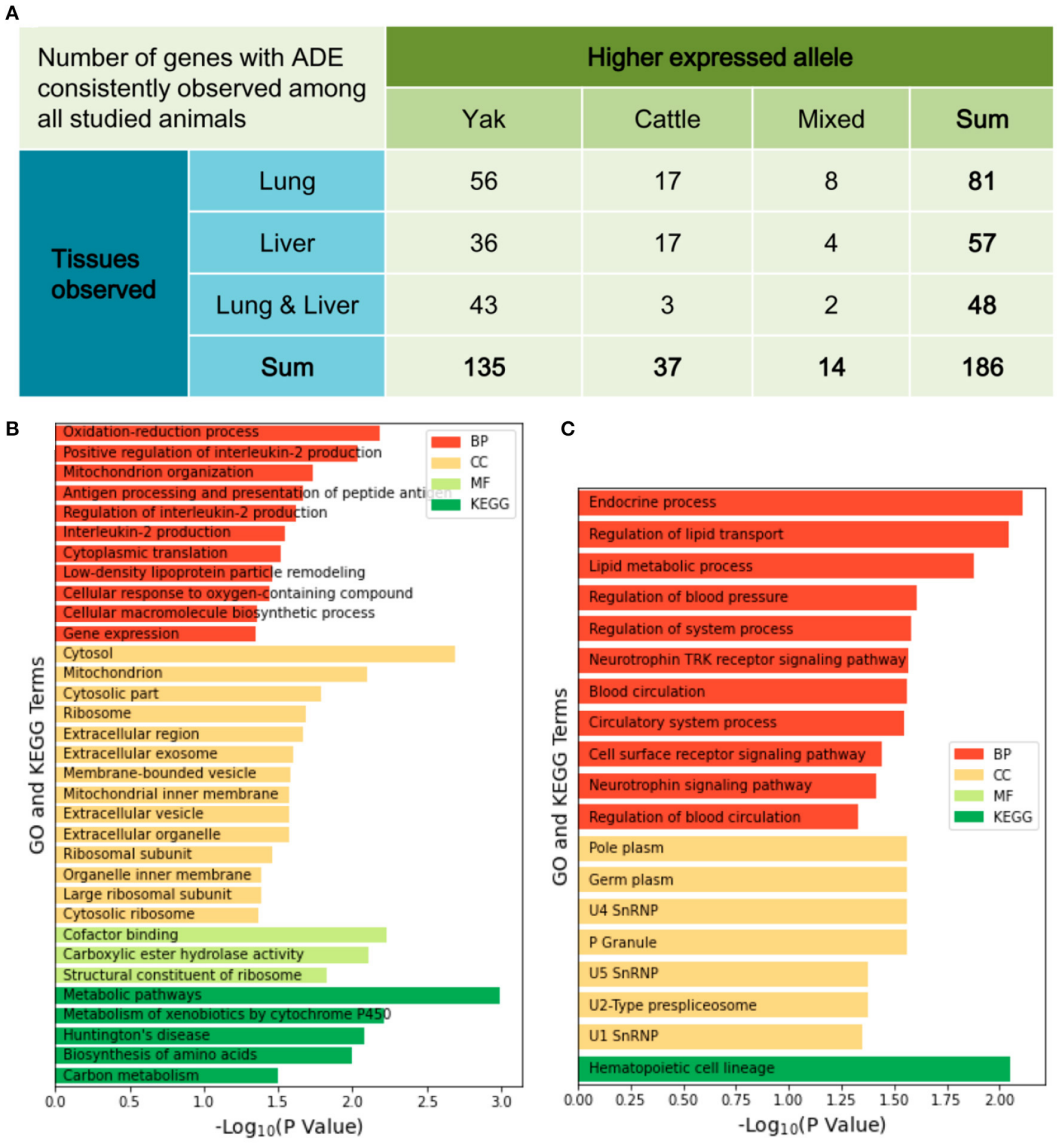
The favoring direction and tissue distribution of allelic differential expression **(A)**. Functional analyses for 135 genes with the consistent higher expression of yak alleles **(B)** and for 35 genes with the consistent higher expression of cattle alleles **(C)**. BP, biological process; MF, molecular function; CC, cellular component; KEGG, Kyoto Encyclopedia of Genes and Genomes.

After quantifying the allelic expression levels, ADE were statistically tested for all the expressed genes in different tissues of each animal. A total of 3,381 genes were found to show ADE in at least one tissue of one animal (Figure 3). Furthermore, 186 genes showed ubiquitous ADE in lung and/or liver tissues among the five biological replications (Figure 4A and Supplementary Table S5). Among them, 135 and 37 genes had the consistent favoring expression of yak alleles and cattle alleles, respectively; whereas 14 genes showed variable direction of favoring expression between parental alleles. Meanwhile, 81 and 57 genes with ADE were specifically found in the lung and liver tissues, respectively; and 48 genes with ADE simultaneously observed in the two tissues.

The 135 genes that show consistent favoring expression of yak allele among all studied animals were significantly enriched into 28 GO terms and five KEGG pathways (Figure 4B). Among them, ten and nine genes were involved in the GO terms of “Oxidation-reduction process” and “Mitochondrion organization,” respectively. Other biologically important functions included the immune responses, change in state or activity of a cell as a result of an oxygen-containing compound stimulus, structural constituent of ribosome, and carbon metabolism. By contrast, the 37 genes showing consistent favoring expression levels of cattle allele were significantly enriched onto 18 GO terms and one KEGG pathway (Figure 4C). Nine and seven genes were involved in the GO terms of “Cell surface receptor signaling pathway” and “Lipid metabolic process,” respectively. Also, the GO terms of “Endocrine process,” “Regulation of blood pressure” and “Blood circulation” were suggested.

## Discussion

Despite the widely acknowledged contribution of gene expression regulation to phenotypic variation, it remains difficult to quantify allelic expression levels mainly because of analytical methodological limitations. However, it is well-known in humans that both *cis*- and *trans*-regulation would differentially affect the two parental alleles and might have significant implications in diverse biological functions (29). Recently, the widespread and dynamic allele-specific expression of immune-related genes were observed among different physiological states of human T cells (30). In farm species, the differential expression of parental alleles have been explored for discovering genetic basis of economically important traits, such as meat quality in cattle (31), disease susceptibility in pigs (32), and heterosis in rice (11). Based on the short RNA-seq reads of 18 tissues from a single cow, Chamberlain et al. (33) reported that about 90% of genes showed ADE in at least one tissue. In this study, we also revealed widespread occurrence of significantly differential expression between parental alleles in the F1 hybrids of cattle and yak.

Using the short-read RNA-seq datasets, Wang et al. (14) reported 883 genes in liver tissues and 592 genes in ear tissues showing significant differential expression between parental alleles in F1 hybrids of cattle and yak; and the long noncoding RNA, pseudo, and lowly expressed genes are more likely to show ADE. According to the direct counting of full-length transcripts from long-read RNA-seq, we found about 20% of the expressed protein-encoding genes showing ADE in at least one sequenced sample, which is higher than the former report of Wang et al. (14); however, such difference would be likely resulted from the different types of sequencing data and analysis methods. There are 186 genes found to show consistent ADE among all the studied animals, which would indicate their important biological implications in F1 hybrids of cattle and yak. As regulatory consequences, the tissue-specific occurrences of ADE have been extensively reported (10, 14, 33, 34). The similar pattern was revealed in the present study as only a small proportion of genes were observed to be simultaneous ADE in both studied tissues. Furthermore, we found that more genes showed ADE in lung tissue than that in liver tissue.

Among the 186 genes showing ubiquitous ADE in all studied animals, we found more genes consistently favoring the expression of yak allele in comparison with cattle allele (N = 135 vs. 37). This result is particularly interesting as it would indicate unequal contribution between parental genes to the F1 hybrids of cattle and yak; and the favoring expression of yak alleles might facilitate environmental adaptation as these animals had been raised in high-altitude environments. Our functional analyses further revealed that these genes have been significantly involved in the biological progresses, such as oxidation-reduction process and mitochondrion organization, that have been previously reported to be associated with hypoxia adaptation and immune response in high-altitude species (35, 36). Among the candidate genes found in this study, for example, the cytochrome b5 type A (*CYB5A*) encodes membrane-bound cytochrome and was found to be one hypoxia-sensitive protein in rat erythrocytes (37); the UDP-glucose 6-dehydrogenase (*UGDH*) gene was involved in the metabolic adaptation to hypoxic stress in human glioblastoma cells (38). In contrast, the genes alternatively favoring expression of cattle alleles have been enriched into different biological progresses, such as the secretion of endocrine hormones and lipid metabolism. For instance, genetic associations with production traits have been reported for the genes of fatty acid binding protein 1 (*FABP1*), dystrophia myotonica protein kinase (*DMPK*), and proopiomelanocortin (*POMC*) in Nanyang, Hanwoo, and Nellore cattle (39–42). On the other hand, the genes with ADE, such as heat shock protein family B member 8 (*HSPB8*), transglutaminase 2 (*TGM2*), and phospholipase A2 group VII (*PLA2G7*), have been also found to play critical regulatory functions in development and maintenance of muscle tissue in Nelore cattle (31). Therefore, we speculate that there would have the biological function-dependent selection pressures regarding the favoring direction of ADE in the F1 hybrids of cattle and yak. However, both *cis*- and *trans*-regulatory variations between parental genomes should be further investigated. Also, it is much interesting to investigate ADE pattern when F1 hybrids of cattle and yak are alternatively raised in low altitude.

To reduce mapping bias of sequencing reads in the F1 hybrids, only homologous regions between the parental genomes were used as reference sequences that account for about 94% of bovine autosome genome. Both inter- and intra-species SNPs were detected and further masked from reference sequence, which is a common strategy for studying the allelic expression (16, 36, 43). Instead of short-read RNA-seq datasets, we employed long sequencing reads in this study that have been proposed to be more robust in terms of assembly of full-length transcripts and quantification of gene expression (20, 44). In high-altitude species, lung and liver are the most important organs in maintaining respiratory and metabolism homeostasis of the entire body, which have been commonly included for studying physiological adaptation to environments [e.g., (3, 14)]. Despite this, one of possible limitations of this study is that only two tissues were analyzed, and muscle tissue should be specifically taken into consideration in future studies (31). Regarding candidate genes found in this study, molecular biology experiments are also required to explore their biological mechanisms influencing the physiological adaptation to environments and the improved production performance for F1 hybrids of cattle and yak.

## Conclusions

In this study, we employed Nanopore long-read RNA-seq technology to quantify genome-wide allelic expression in interspecies F1 hybrids of cattle and yak. Our results revealed many more genes with the favoring expression of yak allele, which would contribute to the physiological adaptation to high-altitude environments. Such unequal contribution of parental alleles will also help us understand the genetic basis of economically important traits in the hybrid animals.

## Data Availability Statement

The raw sequence data reported in this paper have been deposited in the Genome Sequence Archive of Chinese Academy of Sciences (GSA: CRA006347).

## Ethics Statement

Ethical review and approval was not required for the animal study because Biological samples involved in this study were specially collected from commercial slaughterhouse in the Hongyuan County of Sichuan Province.

## Author Contributions

S-YC and CL: conceptualization, formal analysis, and writing—original draft preparation. ZL, XL, and XJ: resources. S-YC, XJ, and S-JL: writing—review and editing. All authors contributed to the article and approved the submitted version.

## Funding

This work was supported by Innovative Research Team of Beef Cattle in Sichuan Province (SCCXTD-2022-13) and Science & Technology Department of Sichuan Province (2021YFYZ0007).

## Conflict of Interest

The authors declare that the research was conducted in the absence of any commercial or financial relationships that could be construed as a potential conflict of interest.

## Publisher's Note

All claims expressed in this article are solely those of the authors and do not necessarily represent those of their affiliated organizations, or those of the publisher, the editors and the reviewers. Any product that may be evaluated in this article, or claim that may be made by its manufacturer, is not guaranteed or endorsed by the publisher.
