# Supplementary material for: Favoring Expression of Yak Alleles in Interspecies F1 Hybrids of Cattle and Yak Under High-Altitude Environments
Source: Front Vet Sci. 2022 Jun 30;9:892663. doi: 10.3389/fvets.2022.892663 (PMC9280030; doi:10.3389/fvets.2022.892663)
Supplement: Supplementary file 1 [file Data_Sheet_1.docx]

**Table S1. Quality information of RNA samples**

| **Tissues** | **Sample ID** | **Concentration (ng/ul)** | **RIN** | **OD260/280** | **OD260/230** |
| --- | --- | --- | --- | --- | --- |
| Lung | F1 | 621.4 | 8.4 | 2.06 | 1.97 |
|  | F2 | 336.8 | 8.3 | 2.06 | 1.75 |
|  | F3 | 897.9 | 8.3 | 2.04 | 1.71 |
|  | F4 | 897.9 | 8.3 | 2.04 | 1.71 |
|  | F5 | 519.4 | 8.7 | 2.06 | 1.95 |
| Liver | G1 | 586.1 | 8.4 | 2.06 | 1.97 |
|  | G2 | 378.4 | 8.4 | 2.06 | 1.72 |
|  | G3 | 786.2 | 8.3 | 2.06 | 1.72 |
|  | G4 | 685.4 | 8.3 | 2.04 | 1.72 |
|  | G5 | 605.2 | 8.6 | 2.04 | 1.89 |

RIN: RNA integrity number

**Table S2. Genome sequencing and identification of interspecies SNPs**

| **Animal ID** | **Clean reads** | **Aligned reads** | **Alignment rate** | **Raw SNPs** | **Interspecies SNPs** |
| --- | --- | --- | --- | --- | --- |
| 1 | 300,081,410 | 292,392,777 | 97.44% | 25,436,108 | 19,314,369 |
| 2 | 312,442,004 | 304,283,986 | 97.39% | 24,908,336 | 19,174,819 |
| 3 | 307,066,952 | 299,177,129 | 97.43% | 25,279,740 | 19,234,004 |
| 4 | 346,104,890 | 336,869,681 | 97.33% | 25,591,478 | 19,802,393 |
| 5 | 307,335,610 | 300,337,383 | 97.72% | 25,321,711 | 19,608,670 |
| **Average** | **314,606,173** | **306,612,191** | **97.36%** | **25,307,475** | **19,426,851** |

**Table S3. Nanopore sequencing results of full-length cDNAs**

| **Tissues** | **Sample ID** | **Raw reads** | **N50 length** | **Reads > 1kb in length** | **Full length reads** | **Mapped reads**  **(%)** |
| --- | --- | --- | --- | --- | --- | --- |
| Lung | F1 | 3,270,933 | 1,459 | 1,481,360 | 2,803,438 | 2,746,326 (97.96%) |
|  | F2 | 3,237,972 | 1,505 | 1,508,910 | 2,851,393 | 2,791,996 (97.92%) |
|  | F3 | 3,448,048 | 1,445 | 1,476,338 | 2,985,234 | 2,930,896 (98.18%) |
|  | F4 | 3,655,444 | 1,420 | 1,623,579 | 3,161,702 | 3,095,182 (97.90%) |
|  | F5 | 4,091,924 | 1,125 | 1,392,998 | 3,680,467 | 3,591,871 (97.59%) |
| Liver | G1 | 3,698,390 | 1,128 | 1,260,068 | 3,238,253 | 3,177,969 (98.14%) |
|  | G2 | 3,724,856 | 1,456 | 1,747,405 | 3,226,936 | 3168365 (98.19%) |
|  | G3 | 3,746,227 | 1,322 | 1,526,165 | 3,322,248 | 3,262,869 (98.21%) |
|  | G4 | 3,837,757 | 1,368 | 1,628,091 | 3,377,173 | 3,329,201 (98.58%) |
|  | G5 | 3,305,683 | 1,493 | 1,551,690 | 2,929,439 | 2,885,887 (98.51%) |

**Table S4. The expressed genes and quantification**

| **Tissues** | **Samples** | **Number of Genes** | **Expression levels (CPM)** | | |
| --- | --- | --- | --- | --- | --- |
|  |  |  | **First quartile** | **Second Quartile** | **Third Quartile** |
| Lung | F1 | 12,474 | 2.628 | 8.215 | 28.26 |
|  | F2 | 12,528 | 2.603 | 8.137 | 29.62 |
|  | F3 | 12,656 | 2.786 | 8.67 | 28.48 |
|  | F4 | 12,860 | 2.336 | 7.885 | 27.74 |
|  | F5 | 12,835 | 2.215 | 6.401 | 21.66 |
| Liver | G1 | 10,698 | 1.735 | 5.207 | 19.38 |
|  | G2 | 10,544 | 1.767 | 5.302 | 21.50 |
|  | G3 | 10,823 | 1.989 | 5.967 | 23.87 |
|  | G4 | 11,509 | 1.915 | 5.198 | 19.97 |
|  | G5 | 10,840 | 1.919 | 5.758 | 23.03 |

CPM: counts per million mapped reads.

**Table S5. The general information of 186 genes with allelic differential expression**

| **Gene symbol** | **Gene name** | **Tissue specificity** | **Favoring allele** |
| --- | --- | --- | --- |
| *LMLN* | leishmanolysin like peptidase | Lungs & Livers | Yak |
| *LOC616200* | glyceraldehyde-3-phosphate dehydrogenase-like | Lungs & Livers | Yak |
| *SLC2A2* | solute carrier family 2 member 2 | Livers | Yak |
| *RPL22L1* | ribosomal protein L22 like 1 | Lungs & Livers | Yak |
| *LOC523130* | C1GALT1-specific chaperone 1 | Lungs & Livers | Yak |
| *COMMD2* | COMM domain containing 2 | Lungs | Yak |
| *GK5* | glycerol kinase 5 | Livers | Cattle |
| *LOC511302* | 40S ribosomal protein S12 | Lungs | Cattle |
| *TFF3* | trefoil factor 3 | Lungs | Yak |
| *POFUT2* | protein O-fucosyltransferase 2 | Lungs | Cattle |
| *EFHB* | EF-hand domain family member B | Livers | Cattle |
| *WDR33* | WD repeat domain 33 | Livers | Yak |
| *CIR1* | corepressor interacting with RBPJ, CIR1 | Lungs | Cattle or Yak |
| *PLA2R1* | phospholipase A2 receptor 1 | Livers | Cattle |
| *KYNU* | kynureninase | Livers | Yak |
| *DARS1* | aspartyl-tRNA synthetase 1 | Lungs & Livers | Yak |
| *UBXN4* | UBX domain protein 4 | Lungs & Livers | Yak |
| *ADAM23* | ADAM metallopeptidase domain 23 | Lungs & Livers | Yak |
| *WDFY1* | WD repeat and FYVE domain containing 1 | Livers | Yak |
| *ITM2C* | integral membrane protein 2C | Lungs | Yak |
| *IFI6* | interferon alpha inducible protein 6 | Lungs | Cattle |
| *SRSF10* | serine and arginine rich splicing factor 10 | Lungs & Livers | Yak |
| *AKR7A2* | aldo-keto reductase family 7 member A2 | Livers | Yak |
| *LOC788425* | aflatoxin B1 aldehyde reductase member 4 | Livers | Yak |
| *CREG1* | cellular repressor of E1A stimulated genes 1 | Lungs | Yak |
| *DUSP23* | dual specificity phosphatase 23 | Livers | Yak |
| *APCS* | amyloid P component, serum | Livers | Yak |
| *NAXE* | NAD(P)HX epimerase | Lungs | Yak |
| *FAM189B* | family with sequence similarity 189 member B | Livers | Yak |
| *HENMT1* | HEN methyltransferase 1 | Lungs | Yak |
| *LOC101907544* | tetratricopeptide repeat protein 9C-like | Lungs | Yak |
| *LOC101906178* | NADH dehydrogenase [ubiquinone] 1 alpha subcomplex subunit 12 | Lungs | Yak |
| *RNF220* | ring finger protein 220 | Lungs | Yak |
| *YBX1* | Y-box binding protein 1 | Lungs & Livers | Yak |
| *LOC782114* | acyl-protein thioesterase 1 | Lungs & Livers | Yak |
| *SAMD9* | sterile alpha motif domain containing 9 | Lungs | Yak |
| *SGCE* | sarcoglycan epsilon | Lungs | Cattle |
| *LOC101902435* | phosphatidylcholine translocator ABCB4-like | Livers | Cattle |
| *IMMP2L* | inner mitochondrial membrane peptidase subunit 2 | Livers | Yak |
| *OPN1SW* | opsin 1, short wave sensitive | Livers | Yak |
| *GIMAP7* | GTPase, IMAP family member 7 | Lungs | Yak |
| *GIMAP6* | GTPase, IMAP family member 6 | Livers | Cattle or Yak |
| *CSRP2* | cysteine and glycine rich protein 2 | Livers | Yak |
| *PPP1R12A* | protein phosphatase 1 regulatory subunit 12A | Lungs & Livers | Yak |
| *NDUFA12* | NADH:ubiquinone oxidoreductase subunit A12 | Lungs | Yak |
| *IGFBP6* | insulin like growth factor binding protein 6 | Lungs | Cattle or Yak |
| *VDR* | vitamin D receptor | Lungs & Livers | Yak |
| *AVPR1A* | arginine vasopressin receptor 1A | Livers | Cattle |
| *LOC524576* | apolipoprotein L3 | Lungs | Yak |
| *SLCO1B3* | solute carrier organic anion transporter family member 1B3 | Lungs | Yak |
| *HEBP1* | heme binding protein 1 | Lungs & Livers | Cattle or Yak |
| *ANO2* | anoctamin 2 | Lungs & Livers | Yak |
| *DDX17* | DEAD-box helicase 17 | Lungs & Livers | Yak |
| *SNU13* | small nuclear ribonucleoprotein 13 | Livers | Yak |
| *C6H4orf3* | chromosome 6 C4orf3 homolog | Lungs & Livers | Yak |
| *MANBA* | mannosidase beta | Lungs & Livers | Yak |
| *DNAJB14* | DnaJ heat shock protein family (Hsp40) member B14 | Lungs & Livers | Yak |
| *TBC1D1* | TBC1 domain family member 1 | Lungs | Cattle |
| *UGDH* | UDP-glucose 6-dehydrogenase | Lungs | Yak |
| *HOPX* | HOP homeobox | Livers | Cattle or Yak |
| *UGT2A1* | UDP glucuronosyltransferase family 2 member A1 complex locus | Livers | Yak |
| *PLAC8* | placenta associated 8 | Lungs | Yak |
| *HSD17B13* | hydroxysteroid 17-beta dehydrogenase 13 | Lungs | Cattle |
| *LOC100849050* | uncharacterized LOC100849050 | Livers | Cattle or Yak |
| *LOC104972780* | collagen alpha-1(I) chain-like | Lungs | Cattle |
| *PLVAP* | plasmalemma vesicle associated protein | Lungs | Cattle |
| *FAM32A* | family with sequence similarity 32 member A | Lungs | Cattle or Yak |
| *NDUFA7* | NADH:ubiquinone oxidoreductase subunit A7 | Lungs & Livers | Yak |
| *HINT1* | histidine triad nucleotide binding protein 1 | Lungs & Livers | Yak |
| *VDAC1* | voltage dependent anion channel 1 | Lungs & Livers | Yak |
| *IK* | IK cytokine | Lungs | Yak |
| *SCGB3A2* | secretoglobin family 3A member 2 | Lungs | Yak |
| *SMIM15* | small integral membrane protein 15 | Lungs & Livers | Yak |
| *MED7* | mediator complex subunit 7 | Lungs & Livers | Yak |
| *ANXA1* | annexin A1 | Lungs & Livers | Yak |
| *TXN* | thioredoxin | Lungs & Livers | Yak |
| *TMEM18* | transmembrane protein 18 | Lungs | Yak |
| *PDSS2* | decaprenyl diphosphate synthase subunit 2 | Lungs & Livers | Cattle |
| *MANEA* | mannosidase endo-alpha | Livers | Yak |
| *HEBP2* | heme binding protein 2 | Livers | Yak |
| *PLAGL1* | PLAG1 like zinc finger 1 | Lungs | Cattle |
| *KIAA1191* | KIAA1191 | Lungs | Yak |
| *LOC100336282* | uncharacterized LOC100336282 | Lungs | Yak |
| *LOC101907658* | 60S ribosomal protein L22-like 1 | Lungs & Livers | Yak |
| *FAM81A* | family with sequence similarity 81 member A | Lungs | Yak |
| *LIPC* | lipase C, hepatic type | Lungs | Yak |
| *RAD51B* | RAD51 paralog B | Lungs | Yak |
| *FOS* | Fos proto-oncogene, AP-1 transcription factor subunit | Lungs | Yak |
| *COX7A2L* | cytochrome c oxidase subunit 7A2 like | Lungs | Yak |
| *SPTBN1* | spectrin beta, non-erythrocytic 1 | Lungs & Livers | Yak |
| *LOC107132928* | endogenous retrovirus group K member 7 Gag polyprotein-like | Lungs | Yak |
| *IL1B* | interleukin 1 beta | Lungs | Cattle |
| *FABP1* | fatty acid binding protein 1 | Livers | Yak |
| *LOC100300483* | antimicrobial peptide NK-lysin | Lungs | Yak |
| *MAT2A* | methionine adenosyltransferase 2A | Lungs & Livers | Yak |
| *POMC* | proopiomelanocortin | Livers | Cattle |
| *CPSF3* | cleavage and polyadenylation specific factor 3 | Lungs & Livers | Yak |
| *ITGB1BP1* | integrin subunit beta 1 binding protein 1 | Lungs | Yak |
| *OR1H1* | olfactory receptor family 1 subfamily H member 1 | Livers | Yak |
| *ESD* | esterase D | Livers | Yak |
| *ANKRD16* | ankyrin repeat domain 16 | Lungs & Livers | Yak |
| *LOC112449294* | dihydrodiol dehydrogenase 3-like | Lungs | Cattle or Yak |
| *CHGB* | chromogranin B | Lungs | Yak |
| *TGM2* | transglutaminase 2 | Lungs | Yak |
| *PLTP* | phospholipid transfer protein | Lungs | Cattle |
| *GSDMC* | gasdermin C | Lungs | Yak |
| *PKIA* | cAMP-dependent protein kinase inhibitor alpha | Lungs & Livers | Yak |
| *VPS13B* | vacuolar protein sorting 13 homolog B | Livers | Yak |
| *DECR1* | 2,4-dienoyl-CoA reductase 1 | Livers | Yak |
| *LOC100848277* | stromelysin-1-like | Lungs | Yak |
| *BUD13* | BUD13 homolog | Lungs | Yak |
| *IFT46* | intraflagellar transport 46 | Livers | Cattle |
| *GALNT18* | polypeptide N-acetylgalactosaminyltransferase 18 | Lungs | Yak |
| *RRM1* | ribonucleotide reductase catalytic subunit M1 | Livers | Yak |
| *CD59* | CD59 molecule | Livers | Cattle |
| *AMBRA1* | autophagy and beclin 1 regulator 1 | Lungs & Livers | Yak |
| *MS4A7* | membrane-spanning 4-domains, subfamily A, member 7 | Lungs | Yak |
| *MYOG* | myogenin | Lungs & Livers | Yak |
| *CR2* | complement C3d receptor 2 | Lungs | Cattle |
| *FMO1* | flavin containing dimethylaniline monoxygenase 1 | Lungs | Yak |
| *PRXL2B* | peroxiredoxin like 2B | Livers | Yak |
| *QSOX1* | quiescin sulfhydryl oxidase 1 | Livers | Yak |
| *HSD11B1* | hydroxysteroid 11-beta dehydrogenase 1 | Livers | Yak |
| *LAMB3* | laminin subunit beta 3 | Livers | Cattle |
| *HSPB8* | heat shock protein family B (small) member 8 | Livers | Cattle |
| *SDSL* | serine dehydratase like | Lungs | Yak |
| *SEC14L3* | SEC14 like lipid binding 3 | Livers | Yak |
| *YWHAH* | tyrosine 3-monooxygenase/tryptophan 5-monooxygenase activation protein eta | Lungs | Yak |
| *LOC508916* | carboxylesterase 1-like | Lungs & Livers | Yak |
| *CCDC113* | coiled-coil domain containing 113 | Lungs | Cattle |
| *POLR2I* | RNA polymerase II subunit I | Lungs & Livers | Yak |
| *CYP2B6* | cytochrome P450 subfamily 2B | Lungs | Yak |
| *DMPK* | dystrophia myotonica protein kinase | Lungs | Cattle |
| *RASIP1* | Ras interacting protein 1 | Lungs | Cattle or Yak |
| *ATF5* | activating transcription factor 5 | Livers | Cattle |
| *KIR3DS1* | killer cell immunoglobulin-like receptor, three domains, short cytoplasmic tail, 1 | Lungs | Yak |
| *LOC107131494* | small nuclear ribonucleoprotein G | Lungs & Livers | Cattle |
| *DUSP14* | dual specificity phosphatase 14 | Lungs | Yak |
| *LOC515676* | keratin-associated protein 5-1 | Lungs | Yak |
| *SLC25A11* | solute carrier family 25 member 11 | Lungs & Livers | Yak |
| *SLC16A11* | solute carrier family 16 member 11 | Livers | Cattle |
| *PRR29* | proline rich 29 | Lungs | Yak |
| *RAB40B* | RAB40B, member RAS oncogene family | Livers | Yak |
| *CDK3* | cyclin dependent kinase 3 | Livers | Yak |
| *NSA2* | NSA2 ribosome biogenesis homolog (S. cerevisiae) | Livers | Yak |
| *TTC33* | tetratricopeptide repeat domain 33 | Lungs & Livers | Yak |
| *CMBL* | carboxymethylenebutenolidase homolog | Livers | Yak |
| *NDN* | necdin, MAGE family member | Lungs | Cattle |
| *SNRPN* | small nuclear ribonucleoprotein polypeptide N | Lungs & Livers | Cattle |
| *LOC100299845* | 60S ribosomal protein L5-like | Lungs & Livers | Cattle or Yak |
| *DNAJA4* | DnaJ heat shock protein family (Hsp40) member A4 | Lungs | Yak |
| *CYP11A1* | cytochrome P450, family 11, subfamily A, polypeptide 1 | Livers | Cattle |
| *LOC617313* | granzyme H | Lungs | Yak |
| *TMEM251* | transmembrane protein 251 | Livers | Cattle |
| *BOLA-DYB* | major histocompatibility complex, class II, DY beta | Lungs | Cattle or Yak |
| *MRPL2* | mitochondrial ribosomal protein L2 | Lungs & Livers | Yak |
| *PLA2G7* | phospholipase A2 group VII | Lungs | Yak |
| *BOLA-DRA* | major histocompatibility complex, class II, DR alpha | Lungs & Livers | Yak |
| *STK19* | serine/threonine kinase 19 | Livers | Yak |
| *LOC781663* | complement C4-A-like | Lungs & Livers | Yak |
| *SFTA2* | surfactant associated 2 | Lungs | Yak |
| *TUBB* | tubulin beta class I | Lungs | Cattle or Yak |
| *RPP21* | ribonuclease P/MRP subunit p21 | Livers | Yak |
| *JSP.1* | MHC Class I JSP.1 | Lungs & Livers | Yak |
| *POLR1H* | RNA polymerase I subunit H | Livers | Yak |
| *CD83* | CD83 molecule | Lungs | Yak |
| *LY86* | lymphocyte antigen 86 | Lungs | Cattle |
| *ECI2* | enoyl-CoA delta isomerase 2 | Lungs | Yak |
| *ZADH2* | zinc binding alcohol dehydrogenase domain containing 2 | Livers | Cattle |
| *CYB5A* | cytochrome b5 type A | Lungs | Yak |
| *C24H18orf21* | chromosome 24 C18orf21 homolog | Lungs & Livers | Yak |
| *LOC100139916* | interleukin 32-like | Lungs | Yak |
| *CARHSP1* | calcium regulated heat stable protein 1 | Livers | Cattle |
| *EMP2* | epithelial membrane protein 2 | Livers | Cattle or Yak |
| *AQP8* | aquaporin 8 | Livers | Yak |
| *SGF29* | SAGA complex associated factor 29 | Lungs & Livers | Yak |
| *PYCARD* | PYD and CARD domain containing | Lungs | Yak |
| *SUMF2* | sulfatase modifying factor 2 | Lungs & Livers | Yak |
| *LOC783012* | beta-defensin-like | Lungs | Yak |
| *TBATA* | thymus, brain and testes associated | Livers | Cattle |
| *RPS24* | ribosomal protein S24 | Lungs | Yak |
| *AGT* | angiotensinogen | Lungs | Cattle |
| *SLC36A4* | solute carrier family 36 member 4 | Lungs | Cattle or Yak |
| *THRSP* | thyroid hormone responsive | Livers | Yak |
| *CCDC85B* | coiled-coil domain containing 85B | Lungs | Yak |
| *PC* | pyruvate carboxylase | Livers | Yak |

**
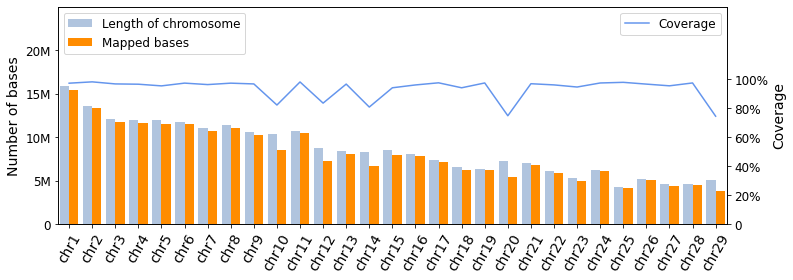
**

**Figure S1 Pairwise alignments of yak genome sequences against 29 autosomes of cattle.**

**
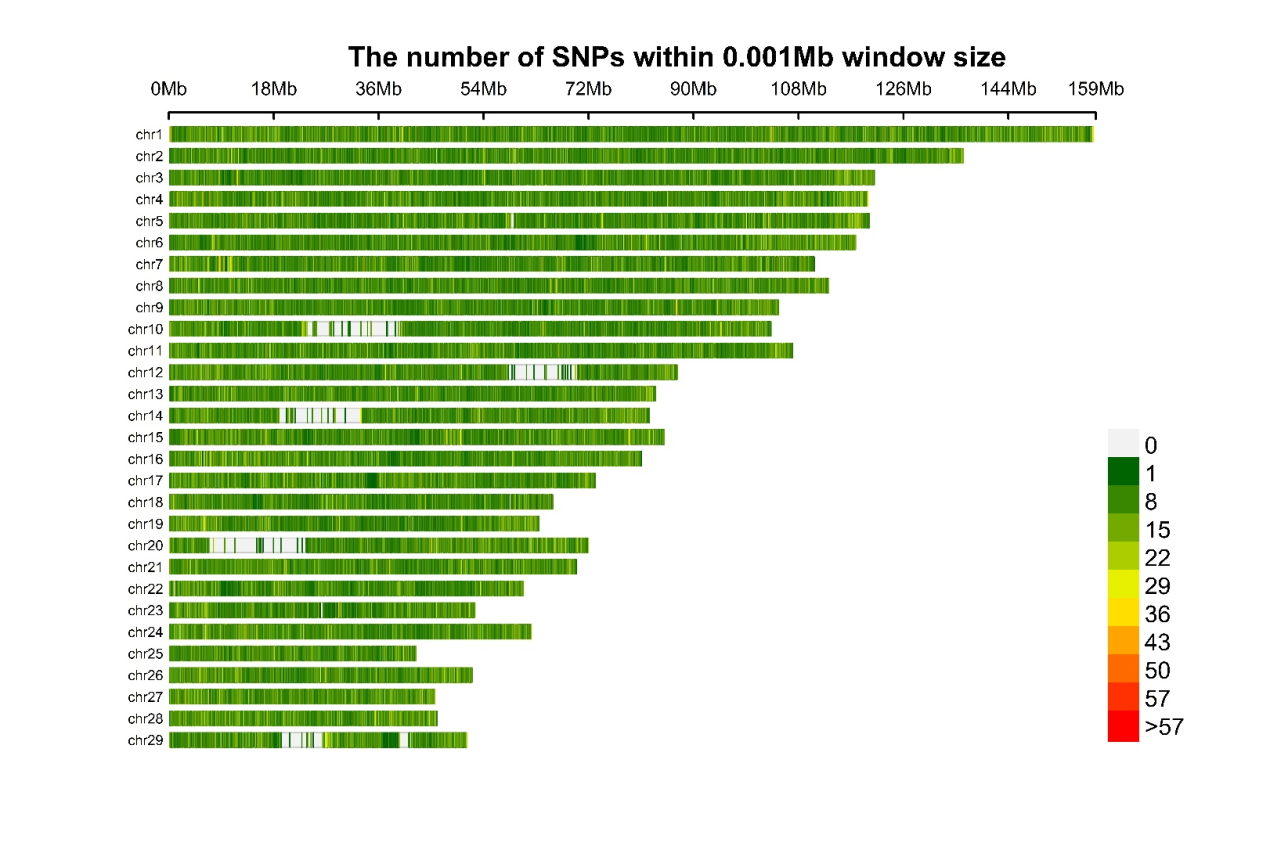
**

**Figure S2 Genomic distribution of the inter-species SNPs detected in the five sequenced animals.**
